# ADME, Pharmacokinetic Scaling, Pharmacodynamic and Prediction of Human Dose and Regimen of Novel Antiviral Drugs

**DOI:** 10.3390/pharmaceutics15041212

**Published:** 2023-04-11

**Authors:** Tridib Chaira, Chandru Subramani, Tarani Kanta Barman

**Affiliations:** 1 Department of Pharmacology, SGT University, Gurugram 122505, Haryana, India; 2 Department of Pathology, Galveston National Laboratory, University of Texas Medical Branch, Galveston, TX 77550, USA

**Keywords:** ADME, allometric scaling, pharmacokinetics-pharmacodynamics, human dose extrapolation, physiological time

## Abstract

The search for new drugs is an extremely time-consuming and expensive endeavour. Much of that time and money go into generating predictive human pharmacokinetic profiles from preclinical efficacy and safety animal data. These pharmacokinetic profiles are used to prioritize or minimize the attrition at later stages of the drug discovery process. In the area of antiviral drug research, these pharmacokinetic profiles are equally important for the optimization, estimation of half-life, determination of effective dose, and dosing regimen, in humans. In this article we have highlighted three important aspects of these profiles. First, the impact of plasma protein binding on two primary pharmacokinetic parameters—volume of distribution and clearance. Second, interdependence of primary parameters on unbound fraction of the drug. Third, the ability to extrapolate human pharmacokinetic parameters and concentration time profiles from animal profiles.

## 1. Introduction

**Figure 1:**
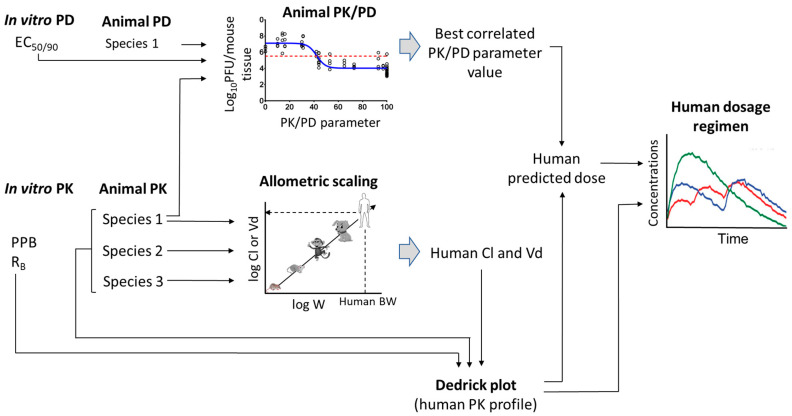
Graphical representation showing critical pathway from in vitro potency test to human dose prediction in discovery process.

Now more than ever, the emergence of deadly viruses and viral pandemics in the last several decades has necessitated the demand for antiviral drug discovery. The discovery and development of new antiviral drugs is one of the most complicated and time-consuming processes which requires a considerable amount of energy and resources. The initial, essential qualification of new chemical entities is in vitro potency. However, the clinical translation with effective efficacy is always challenging due to unfavourable pharmacokinetic (PK) properties and pharmacokinetic-pharmacodynamic (PK-PD) correlation at the pharmacological dose. The process of antiviral drug discovery is summarized graphically in Figure 1. Majority of lead molecules often fail to show the desired efficacy or acceptable safety profiles. The high attrition rates also add to significant increases in resources. Nearly 50% of all drug candidates fail as a consequence of insufficient efficacy [1]—the result of a singular reason, or a combination of several. Nevertheless, the consideration of solubility-permeability relationship helps in the selection of potential oral candidates with desired intestinal absorption. The understanding of absorption, distribution, metabolism, elimination/toxicity (ADME/tox), PK, and calculative prediction of clinical dose at preclinical discovery stage, has ensured the entry of the best antiviral drug candidates into the development phase with optimal ADME properties. Accordingly, more and more attention is drawn toward two main aspects: (i) understanding the correlation and consideration of observed differences in PD/efficacy/safety profiles with PK profiles between animals and humans; and (ii) prediction of human pharmacokinetics at discovery stage and assistance in dialling out potential liabilities in the clinical candidates at an early stage of drug development [2]. The development of oral antiviral drugs is far more complicated and time-consuming compared to parenteral drugs.

The emergence of drug resistance development has outpaced the discovery of new molecules, thus generating a major cause for concern. One significant reason for suboptimal antiviral response could be the result of inadequate exposure and/or poor PK-PD properties of the investigational drug. Maintenance of sufficient plasma exposure within the therapeutic window is one of the most critical requirements to stop viral replication and the emergence of resistance [3]. It has been demonstrated that setting up the dosage regimen based on the PK-PD relationship of antivirals has increased the probability of successful treatment outcome and reduced emergence of resistance [4]. Therefore, the objectives of this paper are: (i) to highlight the importance of plasma protein binding, volume of distribution, and clearance in the antiviral drug discovery process; (ii) to estimate primary PK parameters in humans, such as volume of distribution and clearance from animal data using allometric scaling; (iii) the prediction of human pharmacokinetic profile; and (iv) estimation of the therapeutic dosage and dosage regimen of antiviral drugs using the PK-PD relationship by applying sound mathematical models.

## 2. Role of Volume of Distribution for Antiviral Drug Development

The role of membrane transporters expressed in various tissues in the human body may play an important role in pharmacokinetics, distribution, and toxicity of drugs. Generally, efficacy of a drug has a direct relationship with distribution into tissues of interest. Plasma protein bindings (PPB) have been demonstrated to significantly influence the rate of diffusion of drugs between plasma and tissues. Therefore, PPB influence partitioning between plasma and tissue or volume of distribution at steady state (*V_d,ss_*), and clearance (Cl) of many antiviral drugs, may impact local efficacy [5,6]. The mechanistic role of selective partitioning, tissue binding, and transporters, was appreciated with the uneven distribution of drugs in different tissues following radiolabelled studies in vivo [7,8]. High concentrations of plasma proteins and the inclination of drug molecules to bind with plasma proteins have led drug discovery scientists to think of, and find ways, to modulate the distribution of drug molecules at the target site. Kinetically, the quantitative expression between volume of distribution (*V_d_*), plasma and tissue binding are given by:

(1) $$V_{d}=V_{P}+\sum \left(V_{T}\times \frac{f_{u}}{f_{u,T}}\right)$$

where *f_u_*: unbound fraction in plasma; *V_P_*: plasma volume; *V_T_*: tissue volume; and *f_u,T_*: unbound fraction in tissues.

From Equation (1), it clearly shows *V_d_* is directly proportional to free fraction of drug in plasma and inversely proportional to free fraction of drug in tissue. Drugs with low volume of distribution (*V_d_* < 0.3 L/kg) either have a higher propensity to bind to plasma proteins or are too polar to distribute into tissue. Similarly, high *V_d_* drugs are largely lipophilic and are distributed more into tissues. This implies that a higher dose is required to achieve a necessary concentration in plasma.

Volume of distribution of unbound drugs (*V_u_*) can be shown by rearrangement of Equation (1)

(2) $$V_{u}=\frac{V_{d}}{f_{u}}=\frac{V_{P}}{f_{u}}+\sum \left(\frac{V_{T}}{f_{u,T}}\right)$$

From this equation, it is clear that a change in *f_u,T_* has a greater impact than *f_u_* on *V_u_* because ∑*V_T_* is much greater than *V_P_*.

**Figure 2:**
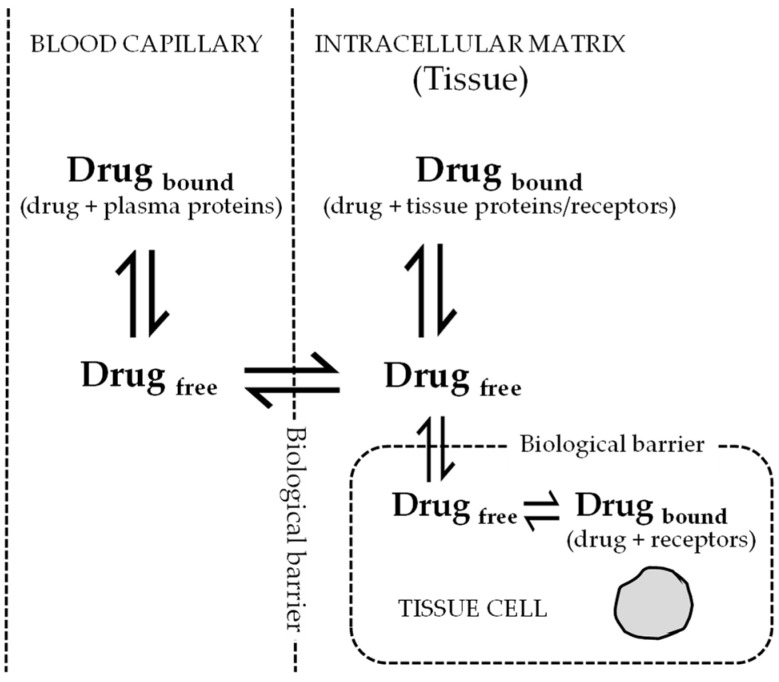
Distribution of unbound (free) drug across membranes at equilibrium. According to free drug theory (FDT), the free drug concentrations across different compartments (blood capillary, intracellular matrix, and tissue cells) are same at equilibrium. The dynamic equilibrium is also maintained between the free drug with the proteins/receptors present in that particular compartment. Only the free drug exerts a pharmacological response when bound to receptor.

As per free drug theory (FDT), after attaining steady state equilibrium, the free drug concentration in plasma is equal to free drug concentration in intracellular spaces of the tissues [9,10]. This theory is widely accepted and explains how PPB of drugs (mainly unbound fraction) relates to pharmacological effects. The total drug concentration in tissues has less or no correlation with the pharmacological effect. A fraction of the unbound drug enters the tissues and binds to tissue proteins; the remaining unbound drug is available for the target receptor binding. If the drug is available at a pharmacologically relevant concentration, it will produce a desired effect. At equilibrium, the free drug concentrations across different tissues are similar and quantitative measurements of unbound drug in plasma will correlate with the free drug in tissues available for binding to cell membrane receptors at the target site (Figure 2). In an in vivo situation, dynamic equilibrium is always maintained between the bound and free drug in a particular compartment. The bound drug or the drug-protein complex becomes too large to diffuse across the cell or capillary membrane. However, the small unbound drug easily diffuses through biological membranes. The diffused free drug binds to proteins present on the opposite side and the equilibrium between bound and free drug is again maintained. Drug concentrations often determined in in vitro samples using chromatography-based methods may not reflect the actual extracellular protein free drug concentrations or intracellular drug concentrations. Both these concentrations may be highly influenced by culture conditions. The error in measurement of extracellular unbound drug concentrations or intracellular drug concentrations is the potential source of discordance between in vitro and in vivo correlations. Within in vivo samples, such as blood or tissue, the drugs bind to various circulating plasma proteins, including albumin, glycoproteins, globulins, and lipoproteins—these may vary across different geographic locations. The concentrations determined in in vivo samples are the total (bound + unbound) drug concentrations [5]. Seeing as only the unbound drug binds to receptors, to exhibit pharmacological effect the estimation of unbound drug concentration may be determined by multiplying the total drug concentration to [1 − (% protein binding/100)]. Unbound drug concentrations in tissues have been commonly used to correlate pharmacokinetic and pharmacodynamic parameters. Antiviral drugs targeted for intracellular viruses (antiretrovirals) exhibit intracellular drug concentrations that are highly influenced by extracellular free drug concentrations.

## 3. Significance Clearance for Antiviral Drug Discovery

Clearance of a drug can be influenced by plasma protein binding. The unbound fraction of the drug is subject to hepatic or renal clearance. Drugs with high plasma protein binding are generally cleared slowly from the body and thereby influence terminal half-life (T_1/2_).

(3) $${Cl}_{hep}=\frac{Q_{H}\times f_{u}\times {Cl}_{int}}{Q_{H}+f_{u}\times {Cl}_{int}}$$

Q*_H_*: hepatic blood flow rate; *f_u_*: unbound fraction in plasma; Cl*_int_*: intrinsic clearance.

Total body clearance is the sum of all organ clearances by which the drug is removed or inactivated by the body—particularly from the renal and metabolic systems.

(4) $${Cl}_{body}={Cl}_{renal}+{Cl}_{hepatic}+{Cl}_{pulmonary}+{Cl}_{etc.}$$

Following intravenous dose, total systemic clearance can be calculated using the formula:

(5) $${Cl}_{total}=\frac{{Dose}_{iv}}{{AUC}_{iv}}$$

Clearance and volume of distribution are both affected by plasma protein binding; thus, also is half-life, as it is dependent upon clearance and volume of distribution by the formula:

(6) T12(h)=ln2×Vd(L)Cltotal(L/h)=0.693×Vd(L)Cltotal(L/h)

Drug transporters—either influx or efflux—play a vital role in disposition of drugs, pharmacological activity, or specific organ-related toxicity. The in vitro and in vivo differences in efficacy of potential test compounds based on in vitro data require initial evaluation for substrates of efflux transporters, mainly P-gp (P-glycoprotein), BCRP (breast cancer receptor proteins), and OATP (organic-anion-transporting polypeptides). These efflux transporters limit absorption from the intestines and drug release to systemic circulation. Alteration of intestinal and/or hepatic transporters by the substrates is one important criterion to be considered in understanding drug-drug interactions (DDIs). Furthermore, the modulation of metabolizing enzymes by genetic polymorphism and diseased condition often causes a change in plasma and/or tissue exposure, leading to change in efficacy and/or toxicological effects [11,12].

## 4. Variation in CYP Mediated Metabolism of Antiviral Drugs

The variability in intestinal absorption and the PK profile in animals and humans is better explained through cytochrome (CYP) P450 mediated metabolism. CYP metabolising enzymes present mainly in the intestine and liver and are important components of drug metabolism and the mechanism that converts drug molecules into a more polar substance—a very complex process. The major substrates and inhibitors of metabolizing enzymes of antiviral drugs are listed in Table 1. Most therapeutic drug interactions observed are due to inhibition and induction of CYP enzymes by different drugs. Genetic variability of CYP isozymes is another important factor for PK variability within species and is a significant source of unpredictable drug effects. Variation in drug response among individuals may reach up to 50% in patients either suffering from low treatment dose or adverse drug reactions. Variability due to genetic factors contribute to around 30% of patients undergoing pharmacotherapy; of these variations in CYP 450 genes, 10–20% are estimated to be of all drug therapies [13]. Out of the 57 CYP enzymes encoded in the human genome, only eight CYP isoforms (CYP2A6, CYP2B6, CYP2C8, CYP2C9, CYP2C19, CYP2D6, CYP3A4, and CYP3A5) are responsible for the biotransformation of most drugs in clinical use [14,15]. Genetic drift and human admixture are the leading factors for this genetic complexity, resulting in the high ethnogeographic differences in genetic variability of CYPs across human populations [16]. The polymorphic forms in three major CYP enzymes, such as CYP2D6, 2C19, and 2C9, are the major cause for variations in phase-1 metabolism of drugs which lead to interindividual variability in efficacy [2,17,18]. Apart from common polymorphisms, a large number of rare variants also add to interindividual variability.

## 5. Scaling of Novel Antivirals from Preclinical to Clinical

There are several aspects of drug development that must be understood before a drug can be tested in humans. In vitro and in vivo animal studies are conducted to understand the efficacy and safety of drug candidates and to provide critical pharmacokinetic information, including ADME properties of the drug. Previously “go/no go” decisions were made on review of nonclinical data, but presently actionable decisions are drawn by critically reviewing the predicted human outcomes along with nonclinical data. This saves time, money, and potential harm to human subject’s reaction to change in efficacy and/or toxicological effects [11,12].

### 5.1. Prediction of Human PK Parameters

Once the decision has been made to advance the potential drug molecule into human studies—or make decisions on lead molecules—nonclinical data are used to predict human outcomes. One common method for predicting human doses is simple allometric scaling [26,27]. Using this technique, preclinical pharmacokinetic parameters, plasma clearance (Cl), and apparent volume of distribution (*V_d_*), are scaled by the exponential function of body weight (BW). Based on the similarities of mammals, a generalized allometric equation can be written as:

(7) $$Y=a\times W^{b}$$

where *Y* is the pharmacokinetic parameter to be calculated, mainly Cl; *W* is the physiological parameter, mainly average body weight of a species; *a* and *b* are allometric coefficient and exponent, respectively. This exponential equation can be made linear after logarithmic transformation and *a* and *b* can be estimated using linear regression. The logarithmic transformation of Equation (7) can be written as:

(8) $$\log\left(Y\right)=\log\left(a\right)+b\times log(W)$$

Plasma clearance of each species is computed via intravenous pharmacokinetic study. The plasma parameter (Cl_plasma_) is converted to blood parameter (Cl_blood_) using R_B_ (blood to plasma partition coefficient ratio) value by the following equation:

(9) $${Cl}_{blood,iv}\left(ml/min\right)=\frac{{Cl}_{plasma,iv}(ml/min/kg)}{R_{B}}\times body\;wt\;\left(kg\right)$$

These intravenous blood pharmacokinetic parameters, from at least three species, are graphically plotted on log(*Y*) versus log(*W*) scale to compute *a* and *b*. Human primary pharmacokinetic parameters are then calculated by using computed *a* and *b* values and average adult human weight. However, for drugs metabolized by the liver, that also have low extraction rate, the error associated in computing these pharmacokinetic parameters using simple allometry is high [26]. It should be taken into consideration that the common practice of scaling of clearance based on body weight (mg/kg) alone may not always give a correct prediction. To minimize prediction errors, the “rule of exponents” (ROE) method is used which provides selection criteria for use of MLP (maximum life span potential) or BrW (brain weight), based on the values of the clearance exponents obtained from simple allometry [28,29,30]. Using this approach, no correction factor is required in predicting human clearance if the value of allometric exponent *b* is between 0.55 and 0.71; MLP should be incorporated into the scaling equation when *b* is between 0.71 and 1.00; BrW should be incorporated when *b* is greater than 1.00. Predicted clearance is underestimated or overestimated if b is greater than 1.30 or less than 0.55, respectively [31].

Human scaling of volume of distribution from animal species is predicted using simple allometry as mentioned in Equation (8) using the Adolph Dedrick method [32].

(10) $$\log\left(\frac{V_{dss,blood,iv}\times R_{B}}{f_{u}}\right)=\log\left(a\right)+b\times log(W)$$

Here *f_u_* is the fraction unbound in plasma. V*_dss,blood,iv_* is volume of distribution at steady state in blood following intravenous administration. The $\left(\frac{V_{dss,blood,iv}\times R_{B}}{f_{u}}\right)$ value in human is then calculated by using *a* and *b* values computed from at least three species and the average adult human weight is estimated. Human V*_dss,blood,iv_* is calculated by multiplying with human (*f_u_*/R_B_). Human predicted elimination half-life is estimated using Equation (6) by incorporating predicted human clearance (Cl) and volume of distribution at steady state (V*_dss_*).

### 5.2. Prediction of Human Plasma-Concentration Time Profiles: Species-Invariant Time Method (Complex Dedrick Plots)

In addition to predicting human pharmacokinetic parameters, prediction of the human plasma concentration-time profile of a drug candidate using modelling and simulation is equally important for predicting PK-PD correlation, estimation of efficacious dose, and dosing frequency in humans.

The general idea behind the physiological species-invariant time method originates from the allometric approach, with the prediction accuracy dependent on the allometric relationships of the pharmacokinetic parameters across species. Short-lived, small animals generally clear drugs from their body at a faster rate (chronological time) per unit of body weight (rate/body weight) compared to long-lived, larger animals. However, if clearing of drugs from the body is measured according to the biological clock of each species, all animals irrespective of different species tend to clear drugs at a similar rate. The species-invariant time method is based on this assumption: by normalizing the concentrations by body weight, and transforming the chronological time to the physiological time, the plasma concentration-time curves should be superimposable in all species [33]. Dedrick first transformed chronological time to the equivalent time, then Boxenbaum introduced new units of pharmacokinetic time: kallynochrons (simple Dedrick) and apolysichrons (complex Dedrick plot); and later introduced apolysichrons and dienetichrons, in which incorporation of maximum life span potential (MLP) and brain weight (BrW) are incorporated in complex Dedrick plot [33,34,35,36]. In the Dedrick plot using equivalent time, the intravenous free plasma concentration-time curve was transformed by dividing the concentrations and time scales of various species by dose (per kilogram of body weight) or W^0.25^, respectively. In the Dedrick plot using kallynochrons, apolysichrons, and dienetichrons, as time units *b* and *c* are the allometric scaling exponents of Cl, V_dss_, and Cl × MLP, respectively [36,37]. The transformed concentration–time curves of various species are superimposed then back-transformed to estimate predicted human intravenous plasma concentration–time profiles at 1 mg/kg dose. The predicted human intravenous profile, along with the estimated mean absorption rate constants and bioavailability, is used to simulate the oral pharmacokinetic profile in humans at 1 mg/kg oral dose. Mahmood and Yuan [37] compared the accuracies of transformations via equivalent time, kallynochrons, and apolysichrons (complex Dedrick plot), for predicting human Cl, *V_dss_*, and *t*_1/2_. The derived complex Dedrick equations are as follows:

(11) $$C_{p}=\frac{D}{V_{d}}\times e^{-\left(\frac{Cl}{V}\right)\times t}$$

(12) $$\frac{C_{p}}{D/W}=\frac{1}{C\times W^{c}/W}\times e^{-\left(\frac{B\times W^{b}}{C\times W^{c}}\right)\times t};\;\left[∵Cl=B\times W^{b},V_{d}=C\times W^{c}\right]$$

B and C are coefficients, and *b* and *c* are the exponents of clearance and volume of distribution, respectively, in simple allometric equation; D is the dose in mg.

(13) $$\frac{C_{p}}{D/W}=\frac{1}{C\times W^{c-1}}\times e^{-\left(\frac{B}{C}\right)\times W^{b-c}\times t}$$

(14) $${C_{p}}^{\prime }=\left(\frac{1}{C\times W^{c-1}}\right)\times e^{-\left(\frac{B}{C}\right)\times t^{\prime }}$$

Concentration scale in y-axis = $={C_{p}}^{\prime }=\left(\frac{C_{p}}{D/W}\right)$ and time scale in x-axis $=t^{\prime }=W^{b-c}\times t$.

## 6. Pharmacokinetic-Pharmacodynamic (PK-PD) of Antiviral Drugs

The understanding of pharmacokinetic and pharmacodynamic (PK-PD) parameters of any drug is key for successful therapy. The concept of PK-PD was initiated in the 1940s by Dr. Harry Eagle [38,39], and later by Dr. William Craig and other investigators in the 1990s. They designed mouse experiments and showed reproducible correlation of animal model and human clinical data [40,41,42]. In today’s current scenario, PK-PD correlation has become increasingly important in the case of anti-infective drugs due to optimal PK-PD parameters’ importance to the reduction in the emergence of resistance. The PK-PD of antibiotics is studied thoroughly and the correlation between preclinical data and human clinical data have been established [40]. The antibacterial activity of antibiotics is either concentration-dependent or time-dependent—activity that is defined by three PK-PD indices. A test tube is used to derive these indices minimum inhibitory concentration (MIC) of antibiotic which completely inhibits the visible growth of microorganisms. MIC is the indicator of potency but does not imply the time course of activity [43]. However, PK shows the time course of antibiotics. The important PK parameters used for PK-PD analysis are area under the concentration-time curve (AUC) and peak concentration or maximum serum concentration (C_max_). The PK parameters indicate the time course of antibiotics but not the antibacterial potency. Therefore, the PK-PD indices, which are indicators of efficacy, are derived by combining both PK parameters and MIC—AUC_0–24h_/MIC, C_max_/MIC, and T_>MIC_ (time above MIC). AUC_0–24h_/MIC is defined as the ratio of area under the concentration-time curve to the MIC; C_max_/MIC is defined as the maximum concentration divided by the MIC; and T_>MIC_ represents the percentage of dosing interval in which drug concentration stays above the MIC. These indices are always calculated as total or free drug concentration based on serum protein binding of the drug [44,45,46,47,48].

Though the concept of PK-PD of antivirals is equivalent to that of antibacterials, the PK-PD of antivirals is not well studied. Antiviral EC_50_ or EC_90_ is an indicator of the antiviral potency of drugs and can be used synonymously as MIC_50_ or MIC_90,_ respectively, of antibacterial. EC_50_ or EC_90_ is defined as the 50% or 90% effective concentration. By integrating the drug exposure to EC_50_ or EC_90_ of the virus (C_max_/EC_50/90_, AUC/EC_50/90,_ and T_>EC50/90_), the clinical antiviral effect can be predicted as the desired pharmacodynamic index [49,50]. The PK-PD studies on commonly available antiviral drugs are scarce. There are four approved antiviral drugs for use against influenza A and B virus infections: oral oseltamivir, inhaled zanamivir, intravenous peramivir, and oral baloxavir. The PK-PD parameters for these drugs are not well defined [51]. These drugs were studied for inhibitory effects on neuraminidase enzyme at different concentrations. Inhibition of viral plaques, cytopathic effects, and viral proteins were studied in cell culture to evaluate the antiviral potency [52,53]. However, the relationship between cell culture inhibition and inhibition of viral replication in human host is not established. The titre of the virus is calculated as plaque-forming units (PFUs). The gold standard for evaluation of antiviral drugs is based on the plaque reduction neutralization test (PRNT). In an animal model, both survival and plaque reduction assays are carried out to assess the PK-PD correlations. The non-availability of sufficient PK-PD parameters on antivirals may be due to several challenges associated with antiviral PK-PD studies. Unlike bacteria, no viruses can be cultured on cell-free media. It requires a sophisticated cell culture facility and trained manpower for antiviral studies. The quantification of viral load in cell culture as well as in infected animal tissues is a tedious process. The EC_50_ value of oseltamivir carboxylate against influenza A and B strains were reported to be 0.17–44 μg/L in cell culture [51]. The pharmacodynamic studies of these inhibitors in the form of an in vivo efficacy study in ferret and mouse were carried out which supported the inhibitory activity observed in the in vitro system [54]. Five-day treatment of oseltamivir protected mice from influenza A and B virus infection and reduced lung viral burden against influenza A virus infection [53]. However, PK-PD parameters predictive of in vivo efficacy are yet to be ascertained. In one clinical trial, 75 mg and 150 mg, twice-a-day dosage, exhibited a similar clinical outcome [52]. The PK/PD study of oseltamivir was also studied in the hollow fibre model. The AUC_0–24h_ to IC_50_ ratio was predicted as the pharmacodynamic parameter of efficacy in the hollow fibre model [55]. Similarly, AUC/EC_50_ was found to be the PK-PD parameter of peramivir in the mouse model of influenza [56]. However, PK-PD index for intravenous zanamivir was not AUC/EC_50,_ but T_>EC50_ [57]. The terminal half-life also plays an important role in predictive PK-PD parameters in the same class of drugs. For example, oseltamivir and zanamivir have a terminal half-life of 8 h and 2.5 h, respectively. In contrast to T_>EC50_ of zanamivir, oseltamivir exhibited AUC_0–24h_/EC_50_ as the best predictor of efficacy linked to half-life. Though these studies provide excellent understanding into PK-PD indices of antiviral drugs, more clinical data are needed to make more robust PK-PD modelling of antiviral drugs.

### Prediction of Human Efficacy Dose Using Different PK-PD Parameters

**Figure 3:**
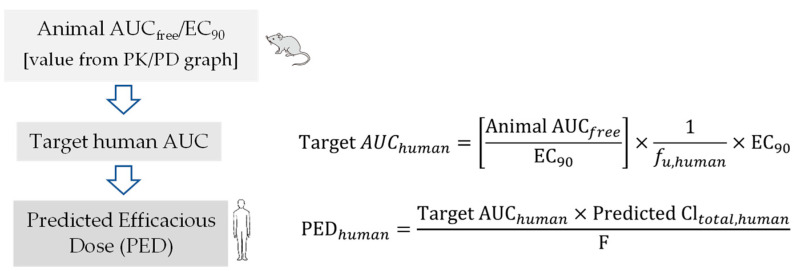
Flow chart for prediction of human efficacious dose using animal AUC_free_/EC_90._ Animal AUC_free_/EC_90_ value is estimated from PK/PD graph. Human PED is estimated using calculated Target human AUC value using above mathematical expression.

**Figure 4:**
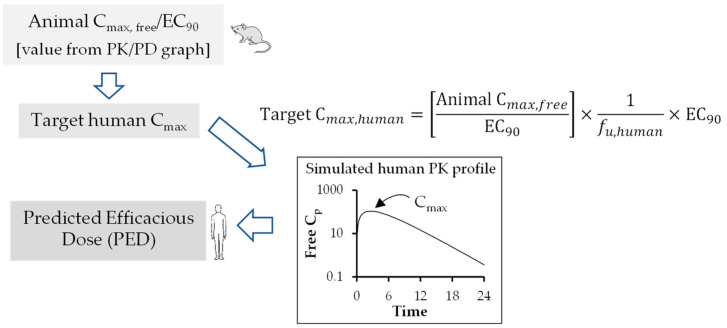
Flow chart for prediction of human efficacious dose using animal C_max,free_/EC_90._ Animal C_max,free_/EC_90_ value is estimated from PK/PD graph. Target human C_max_ is calculated from the above mathematical expression. The human PED is estimated from the simulated free Cp versus time curve. The dose at which the Cmax of the simulated free Cp versus time curve coincides with Target human Cmax is the human PED.

**Figure 5:**
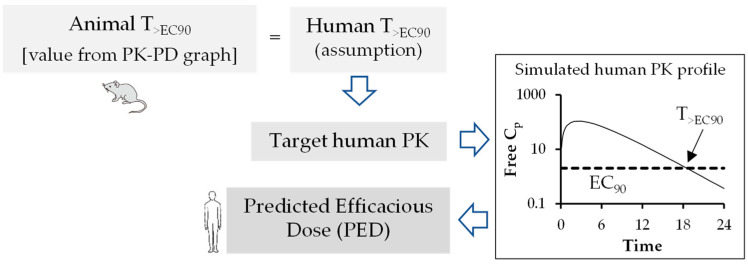
Flow chart for prediction of human efficacious dose using animal T_>EC90._ Animal T_>EC90_ value is estimated from PK/PD graph. The human PED is estimated from the simulated free Cp versus time curve with EC_90_ level. The dose at which the simulated free Cp versus time curve intersects with the animal T_>EC90_ value is the human PED.

An optimal PK-PD index is established for antivirals using the graphical representation of PK-PD analysis similar to antibacterial drugs. The human efficacious dose can be predicted using preclinical animal PK-PD parameters such as AUC_free_/EC_50/90_ (Figure 3), C_max, free_/EC_50/90_ (Figure 4) and T_>EC50/90_ (Figure 5). Figure legend of each figure explains how they are calculated using mathematical expression. Both EC_90_ and EC_50_ values can be used for calculation of target AUC in human. However, EC_90_ is prefrred over EC_50_ for adequate coverage ratio. 

## 7. Conclusions

In summary, during the preclinical phase of the drug discovery process, prior information on human pharmacokinetic parameters such as Cl, V_d_, and t_1/2,_ provided tremendous value in the compound selection process and simulation of human pharmacokinetic profile from predicted human pharmacokinetic parameters for prediction of dosage regimen. Therefore, in recent years, interspecies scaling of pharmacokinetic parameters and prediction of human pharmacokinetic profile using PK-PD analysis have drawn enormous attention in antiviral drug discovery. However, more robust pharmacodynamic data in the form of reduction in PFUs are to be generated in cell culture and animal models to best correlate the PK-PD parameters. This will help overcome the challenges associated with PK-PD correlations. Two important pharmacokinetic parameters, such as clearance and volume of distribution, are required to simulate time course of drug profiles in humans and to predict human t_1/2_, a parameter that is better understood by non-pharmacokineticist colleagues in the drug discovery and development field. Estimation of human half-life using predicted human clearance and volume of distribution generally provides more acceptable results in predicting t_1/2_ rather than direct correlation of animal and human t_1/2_ values [58]. Over the years, multiple approaches have been suggested to improve the predictive performance of time course profiles, however, there is no method without shortcomings [59]. Hence, consideration of a particular extrapolation method should be conducted based upon physicochemical properties of drugs such as renal secretion or biliary excretion. To improve predictive outcome, careful attention should be given to experimental design, choice of in vivo study species, and analytical errors. In addition, all these factors may have an impact on allometric extrapolation. The animal scaling method for prediction of time course of drug profiles using complex Dedrick plot and human therapeutic dose using various rational PK-PD models (AUC/EC_50/90_, C_max_/EC_50/90,_ and T_>EC50/90_) mentioned in this article are simplified, reliable, and a relatively less time-consuming method for extrapolation of preclinical data to humans.

## Figures and Tables

**Table 1:** Major substrates and inhibitors of antivirals.

Antiviral Class [Reference]	Drug	Substrate of	Inhibitor of	BCS Class
Hepatitis C antiviral [19]	Simeprevir	CYP3A4	CYP3A4/1A2	IV
	Ombitasvir	CYP2C8	UGT1A1	IV
	Paritaprevir	CYP3A4/3A5	UGT1A1	IV
	Boceprevir	CYP3A4/3A5	CYP3A4/3A5	IV
	Dasabuvir	CYP2C8/3A4	UGT1A1	IV
Protease inhibitor [20]	Indinavir	CYP3A4, UGT1A1	CYP3A4	II/IV
	Nelfinavir	CYP2C19/3A4	CYP3A4	IV
	Saquinavir	CYP3A4	CYP3A4	IV
	Tipranavir	CYP3A4	CYP3A4/2D6	II
	Darunavir	CYP3A4	CYP3A4	II
	Lopinavir	CYP3A4	CYP3A4 (minor)	IV
	Fosamprenavir	CYP3A4	CYP3A4	II
	Ritonavir	CYP3A4/2D6	CYP3A4/2D6 (minor)	IV
	Atazanavir	CYP3A4	CYP2C8/3A4 (minor)	II
NRTIs [21]	Tenofovir disoproxil fumarate	Not CYP substrate	Not CYP inhibitor	III
	Abacavir	Not CYP substrate, HLA-B	Not CYP inhibitor	III
	Zidovudine	hepatic glucuronidation, UGT	Not CYP inhibitor	I
	Lamivudine	OCT1, OCT2	Not CYP inhibitor	III
NNRTIs [20]	Efavirenz	CYP2B6 (major)/2A6/3A4	CYP3A4	II/IV
	Nevirapine	CYP3A4/2B6	Not CYP inhibitor	II
	Etravirine	CYP3A4/2C9/2C19	CYP2C9/2C19	IV
	Rilpivirine	CYP3A4	Not CYP inhibitor	II
	Delavirdine	CYP3A4	Not CYP inhibitor	III
HIV integrase inhibitors [20,21,22,23]	Dolutegravir	3A4 (minor), UGT1A1	Not CYP inhibitor	II
	Elvitegravir	CYP 3A4	Not CYP inhibitor	II
	Raltegravir	Not CYP substrate	Not CYP inhibitor	II
Nucleotide polymerase inhibitors [24]	Sofosbuvir	CYP 3A4/3A5	Not CYP inhibitor	III
CCR5 antagonist [25]	Maraviroc	CYP3A4/3A5	Not CYP inhibitor	III

NRTIs: nucleoside reverse transcription inhibitors; NNRTIs: non-nucleoside reverse-transcriptase inhibitors.

## Data Availability

Not applicable.
